# Viscoelastic Hemostatic Assays: A Primer on Legacy and New Generation Devices

**DOI:** 10.3390/jcm11030860

**Published:** 2022-02-07

**Authors:** Oksana Volod, Connor M. Bunch, Nuha Zackariya, Ernest E. Moore, Hunter B. Moore, Hau C. Kwaan, Matthew D. Neal, Mahmoud D. Al-Fadhl, Shivani S. Patel, Grant Wiarda, Hamid D. Al-Fadhl, Max L. McCoy, Anthony V. Thomas, Scott G. Thomas, Laura Gillespie, Rashid Z. Khan, Mahmud Zamlut, Peter Kamphues, Dietmar Fries, Mark M. Walsh

**Affiliations:** 1 Department of Pathology and Laboratory Medicine, Cedars-Sinai Medical Center, Los Angeles, CA 90048, USA; oksana.volod@cshs.org; 2 Department of Internal Medicine, Saint Joseph Regional Medical Center, Mishawaka, IN 46545, USA; cmbunch@iu.edu (C.M.B.); malfadhl@iu.edu (M.D.A.-F.); ssp@iu.edu (S.S.P.); grwiarda@gmail.com (G.W.); halfadhl@iu.edu (H.D.A.-F.); maxmcco@iu.edu (M.L.M.); 3 Department of Internal Medicine, Indiana University School of Medicine South Bend Campus, Notre Dame, IN 46617, USA; nzackari@iu.edu (N.Z.); anvthoma@iu.edu (A.V.T.); 4 Department of Surgery, Ernest E. Moore Shock Trauma Center at Denver Health and University of Colorado Health Sciences Center, Denver, CO 80204, USA; Ernest.moore@dhha.org (E.E.M.); hunter.moore@ucdenver.edu (H.B.M.); 5 Division of Hematology and Oncology, Department of Medicine, Northwestern University Feinberg School of Medicine, Chicago, IL 60611, USA; h-kwaan@northwestern.edu; 6 Pittsburgh Trauma Research Center, University of Pittsburgh Medical Center, Pittsburgh, PA 15213, USA; nealm2@upmc.edu; 7 Department of Trauma Surgery, Memorial Leighton Trauma Center, Beacon Health System, South Bend, IN 46601, USA; sthomas@beaconhealthsystem.org; 8 Department of Quality Assurance and Performance Improvement, Saint Joseph Regional Medical Center, Mishawaka, IN 46545, USA; gillesla@sjrmc.com (L.G.); peter.kamphues@sjrmc.com (P.K.); 9 Department of Hematology, Michiana Hematology Oncology, Mishawaka, IN 46545, USA; rkhan@mhopc.com; 10 Department of Critical Care Medicine, Saint Joseph Regional Medical Center, Mishawaka, IN 46545, USA; mzamlut@gmail.com; 11 Department of Anesthesiology and Critical Care Medicine, Medical University of Innsbruck, 6020 Innsbruck, Austria; dietmar.fries@i-med.ac.at; 12 Department of Emergency Medicine, Saint Joseph Regional Medical Center, Mishawaka, IN 46545, USA

**Keywords:** thromboelastography, rotational thromboelastometry, COVID-19, coagulopathy, hemorrhage, thrombosis, personalized medicine, fibrinogen, heparin

## Abstract

Viscoelastic hemostatic assay (VHAs) are whole blood point-of-care tests that have become an essential method for assaying hemostatic competence in liver transplantation, cardiac surgery, and most recently, trauma surgery involving hemorrhagic shock. It has taken more than three-quarters of a century of research and clinical application for this technology to become mainstream in these three clinical areas. Within the last decade, the cup and pin legacy devices, such as thromboelastography (TEG^®^ 5000) and rotational thromboelastometry (ROTEM^®^ delta), have been supplanted not only by cartridge systems (TEG^®^ 6S and ROTEM^®^ sigma), but also by more portable point-of-care bedside testing iterations of these legacy devices (e.g., Sonoclot^®^, Quantra^®^, and ClotPro^®^). Here, the legacy and new generation VHAs are compared on the basis of their unique hemostatic parameters that define contributions of coagulation factors, fibrinogen/fibrin, platelets, and clot lysis as related to the lifespan of a clot. In conclusion, we offer a brief discussion on the meteoric adoption of VHAs across the medical and surgical specialties to address COVID-19-associated coagulopathy.

## 1. Introduction: The Long History of Viscoelastic Hemostatic Assaying in Research and Clinical Medicine

Introduced in 1948 as a research tool to describe the hemostatic competence of whole blood, the viscoelastic hemostatic assay (VHA) thromboelastography (TEG^®^) first entered the operating theater in the late 1960s. It was here that TEG^®^ began its first routine use for liver transplantations in guiding blood component therapy (BCT) and hemostatic adjunct therapy (HAT), such as antifibrinolytics and heparin reversal with protamine sulfate [1,2,3,4,5,6]. Beyond liver transplantation and cardiac surgery, TEG^®^ adoption was nearly stagnant through the first decade of the 21st century. In recent years, TEG^®^ has also been adopted for guiding trauma resuscitation in a goal-directed fashion. Around the same time, in the early 21st century, rotational thromboelastometry (ROTEM^®^) technology was also used successfully in similar areas [5,7,8,9,10,11,12].

Since the 1960s, the delay in the wide acceptance of TEG^®^ and ROTEM^®^ has largely been a function of the lengthy process of confirmation by large randomized controlled trials (RCTs) and Cochrane analyses. Specifically, the clinical value of the VHAs in question includes their point-of-care (POC) utility in the following settings: liver transplantation, cardiac surgery, trauma, obstetrical bleeding, and, most recently, severe hemorrhage in the medical intensive care unit [13,14,15,16,17,18,19,20].

Traditionally, blood banking specialists and hematologists have typically used plasma-based coagulation tests to assess bleeding and clotting risks in surgical, trauma, and non-trauma patients. However, such tests were initially developed to assess patients with isolated clotting factor deficiencies or who were being treated with anticoagulant therapy. These common coagulation tests (CCTs) included the international normalized ratio (INR), prothrombin time (PT), activated partial thromboplastin time (aPTT), platelet counts, levels of fibrinogen, D-dimer, and anti-Xa levels. These tests were subsequently extrapolated for utilization in diverse clinical scenarios.

The CCTs’ utility in evaluating severely hemorrhaging and septic patients has been questioned with the advent of the cell-based model of hemostasis, supported by the CCTs’ lack of sensitivity and slow turnaround time. These shortcomings have prompted trauma surgeons, anesthesiologists, and most recently, obstetricians and intensivists, to turn to VHA-guided BCT for evaluating their bleeding patients [1,3,8,9,16,17,21,22,23,24,25]. For the reasons mentioned above, there has been an increased interest in the use of VHAs to guide BCT by a broad range of medical and surgical specialties. Summarized below are the principles and uses of the most common VHAs in the research and the clinical arenas.

## 2. Principles of Viscoelastic Hemostatic Assays (VHAs)

The term “viscoelasticity” refers to the property of certain materials to exhibit both viscous and elastic characteristics when undergoing deformation. Blood is a viscous material under normal conditions. However, during the process of coagulation, blood experiences radical changes that cause it to lose viscosity and become more elastic in nature. Complex clot structure enables clots to resist deformation under shear forces. The ability of a material to resist deformation is called shear modulus (G), or modulus of rigidity, and is a measure of the elastic shear stiffness of a material. With VHA systems, even though the term “clot strength” is used frequently, clot stiffness is measured either indirectly or directly. Using TEG^®^ 5000 and ROTEM^®^ delta, shear modulus is assessed indirectly by submerging a pin in whole blood, whereas in Quantra^®^, it is assessed directly with a sonographic method. The TEG^®^ 5000 and ROTEM^®^ delta “cup and pin” technologies are referred to as “legacy devices” [26,27].

### 2.1. Thromboelastography (TEG^®^ 5000)

**Figure 1:**
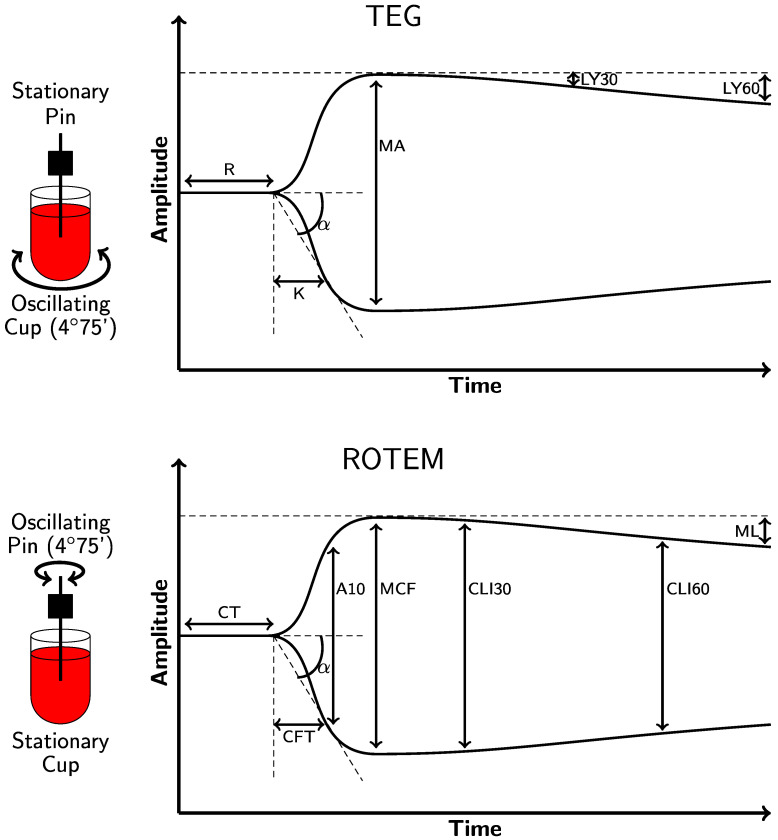
Depictions of the physiologic TEG^®^ 5000 (**top**) and ROTEM^®^ delta (**bottom**) tracings. As the left side of the figure illustrates, a pin descends into a cup containing a sample of whole blood that is maintained at 37 °C. TEG^®^ 5000 and ROTEM^®^ delta analyzers use equivalent parameters that are labeled differently. Reaction time (R) and clotting time (CT) measure the time required for the transducer to displace by 2 mm on the y-axis. Clot formation/kinetics (K) and clot formation time (CFT) measure the initial clot strength and the time needed to displace the transducer by 20 mm, measured from when it first reached 2 mm. The α-angle measures the rate of clot formation in both the TEG^®^ 5000 and ROTEM^®^ delta analyzers by analyzing the angle formed between the end of the R/CT (which is called the split point) and the 20 mm point on the y-axis. The fibrinogen level is broadly correlated with both the K/CFT and α-angle. The reference ranges and definitions of each parameter are provided in Table 1. The clot amplitude at 5 and 10 min (A5 and A10) measures the amplitude at 5 min intervals after the end of R/CT. The maximum amplitude (MA) and maximum clot firmness (MCF) measure the maximum displacement and are indicative of the maximum clot strength. They also correlate with the maximum clot retraction and reflect the crosslinking of fibrin with platelets. Fibrinolysis is depicted by differing parameters in the TEG^®^ 5000 and ROTEM^®^ delta analyzers. The lysis at 30 and 60 min (LY30 and LY60) is a measure of the percent of decrease in amplitude at 30 and 60 min after achieving MA. The clot lysis index (CLI30 and CLI60) is the residual clot remaining 30 and 60 min after CT measured as a percentage of MCF. The maximum lysis (ML) is a measure of the percent of decrease in amplitude at the end of the run [20,23,34,35,36,37,38,39,40,41].

For the TEG^®^ 5000 (Haemonetics Corporation, Boston, MA, USA), a 0.36 cc sample of whole blood is placed into a cup which rotates at 4.45 degrees every 10 s. Blood is recalcified and activated with activators. A pin is placed into this cup, and as a clot forms between the rotating cup and the stationary pin, a force is transmitted to the pin. This force is measured on a computer as an output where the x-axis represents the time in minutes, and the y-axis represents the movement of the pin in millimeters. The TEG^®^ 5000 parameters include the reaction time (R), clot formation/kinetics (K), α-angle, maximum amplitude (MA), and lysis at 30 min (LY30) (Figure 1 and Table 1) [28,29,30,31]. In addition, when quicker results are necessary, such as with cardiac surgery, hypotensive resuscitation during trauma, and liver transplantation, the addition of tissue factor (TF) to the cup is defined for TEG^®^ as RapidTEG^®^ (rTEG^®^) [32,33,34].

### 2.2. Rotational Thromboelastometry (ROTEM^®^ Delta)

With the ROTEM^®^ delta (Instrumentation Laboratory, Bedford, MA), the cup remains stationary while the pin revolves. This results in a curve similar to that of the TEG^®^ 5000, based on similar rheological principles but with different parameters and terminology (Figure 1 and Table 1). In liver transplantation, cardiac surgery, postpartum hemorrhage, and trauma resuscitation, ROTEM^®^ delta has been demonstrated to be effective for goal-directed BCT. ROTEM^®^ has also been used in the diagnosis of bleeding disorders. Extrinsic thromboelastometry (EXTEM) as a parameter of ROTEM^®^ delta, modified by the addition of TF to the cup, provides “interim results” much the same as the TF-enhanced rTEG^®^ produces threshold parameters that trigger action by the clinician prior to the completion of the test in emergency situations [11,29,32,37,53,54].

### 2.3. Chemical Activators and Inhibitors of VHAs and Their Interpretation

The TEG^®^ 5000 and ROTEM^®^ delta parameters have general similarities. For example, EXTEM and rTEG^®^ both provide 5 min and 10 min interval evaluations of the width of the curve from the split point defined as CA5 and CA10 in EXTEM and A5 and A10 in rTEG^®^. These values reflect the addition of the TF-enhanced width of the clot from the split point until it reaches MCF for the EXTEM or MA for the rTEG^®^ as a “real-time” reflection of the maximum clot firmness. Therefore, clinical specialists who are responsible for the bedside monitoring of the hemostatic competence of patients can better anticipate blood component needs with greater accuracy and speed [11,32,34,55,56].

Different chemical activators and inhibitors are used by the TEG^®^ 5000 and ROTEM^®^ delta analyzers, allowing for the depiction of the mechanisms of coagulation. In addition, the thresholds for intervention are not uniform and depend on local standards that determine the triggers for intervention [13,36,57]. VHA guidance of BCTs, and pro-hemostatic agents with simplified algorithms, facilitate goal-directed BCT and HAT for bleeding and anticoagulated patients [44].

By adding different coagulation enhancers or inhibitors to the cup, modified TEG^®^ 5000 or ROTEM^®^ delta analysis allows for more detailed analysis of specific coagulation mechanisms, including fibrinolysis and the effect of heparin on clot strength (Table 2). For example, to assess hypocoagulopathy in the operating theater or in the emergency department, tissue factor is used to run the rTEG^®^ or EXTEM [32].

### 2.4. Sonoclot^®^

The TEG^®^ 5000 and the ROTEM^®^ delta systems are considered cup and pin “legacy” systems. Another legacy linear motion system is called Sonoclot^®^; however, it is not a rotation-based system. An empty plastic probe is attached to the transducer head and submerged into an assay sample with potentially varying clotting factors. The oscillation of the probe within the sample transmits a force to a transducer which emits a signal that is proportional to the deflection of the tube. The oscillatory patterns of the movement of the test probe directly reflect the viscoelastic properties of the blood sample. On the x-axis, time is marked, and on the y-axis, millimeters of the movement of the test probe are marked, similar to the function of the TEG^®^ 5000 and ROTEM^®^ delta [27,61].

### 2.5. New Clinical Technology for VHAs

#### 2.5.1. TEG^®^ 6s

**Figure 2:**
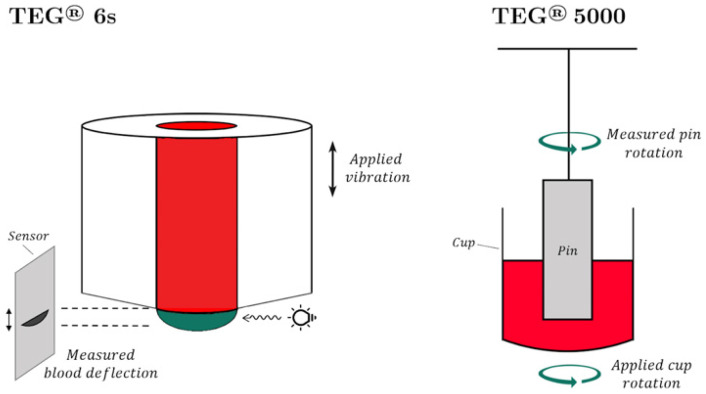
(**Left**): TEG^®^ 6s. The vibration of the sample induces blood deflection; the measures change with the resonance frequency. (**Right**): TEG^®^ 5000. The difference in the rotations of the cup and pin is measured via a torsional spring [64,65].

The TEG^®^ 6s Hemostasis Analyzer (Haemonetics Corporation, Boston, MA, USA) is a cartridge-based system which confronts the problem of operator-dependent variability in the pipetting technique [62]. The TEG^®^ 6s cartridge system measures the resonance frequency of a whole blood sample that is exposed to frequency vibrations caused by the motion of the blood meniscus. The resulting resonance frequency of the whole blood sample is measured by illuminating the sample with a light-emitting diode (LED). As the clot forms and the alteration of resonance is measured by the LED, the collected data is converted into a graph that is identical to that used in the cup and pin method (Figure 2) [36,63].

The TEG^®^ 5000 and the TEG^®^ 6s devices have demonstrated a high degree of reliability, close correlation, and the equivalency of results with the exception of LY30 [66]. Differences in equivalency regarding the manufacturer’s (Haemonetics Corporation, Boston, MA, USA) reference range values between TEG^®^ 6s and TEG^®^ 5000 can be associated with the CLSI methodology used for their TEG^®^ 6s ranges. It has also been suggested that the TEG^®^ 6s offers a significant advantage as a point-of-care test to determine hemostatic integrity [67]. Currently, three TEG^®^ 6s cartridges are Food and Drug Administration (FDA) approved in the United States for adult patients: global hemostasis with lysis, global hemostasis, and the Platelet Mapping adenosine diphosphate (ADP) and arachidonic acid (AA) cartridge. Each has different indications and assay combinations. Although the TEG^®^ 6s and the TEG^®^ 5000 use slightly different mechanisms to assess similar coagulation components, there is a small difference in absolute values of respective parameters of the two devices [62,66,67]. Therefore, the absolute values are not interchangeable. The definition and clinical significance of TEG^®^ 6s parameters are detailed in Table 3 [27].

#### 2.5.2. ROTEM^®^ Sigma

The ROTEM^®^ sigma device is a completely automated device which has replaced the practice of pipetting in favor of inserting a tube of blood directly into the instrument. This technology also uses the pin and cup technique similar to the ROTEM^®^ delta. This allows for the use of the same algorithms that are already established for the ROTEM^®^ delta device. Recent papers have been published by teams in Europe approving its comparability with the ROTEM^®^ delta device. Currently, the FDA validation for the specific use of A5 in the USA is pending [12,59,75,76,77]. The definition and clinical significance of the ROTEM^®^ sigma parameters are detailed in Table 3.

#### 2.5.3. Quantra^®^

**Figure 3:**
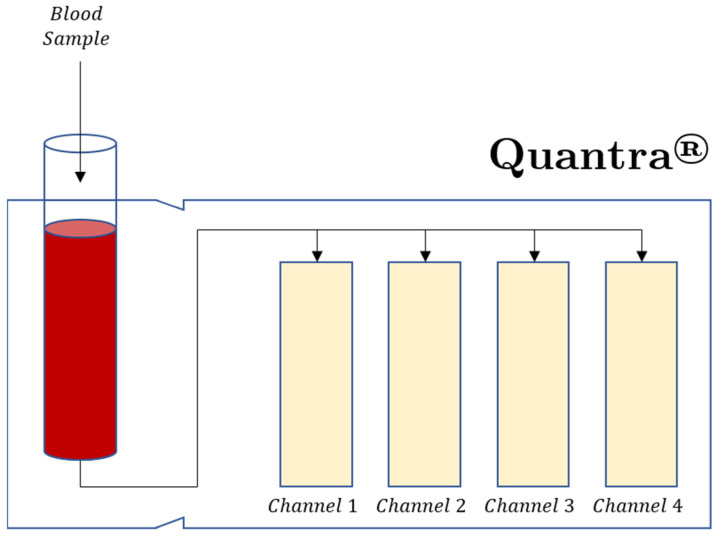
The Quantra^®^ plus cartridge has four channels with reagents designed to measure clot stiffness (CS) and clot time (CT). Channel 1 measures CT with kaolin activation. Channel 2 measures CT with kaolin activation and heparin neutralization. Channel 3 measures CS and tissue factor activation with heparin neutralization. Channel 4 measures CS with tissue factor activation, platelet inhibition, and heparin neutralization. Channel 4 measures the fibrinogen contribution to CS, (FCS). The platelet contribution to CS, (PCS) is equal to CS-FCS. Clot Time Ratio (CTR) = CT/CTH and indicates the level of heparinization of the patient blood sample [69,78].

Newer bedside POC VHAs have also recently been reviewed, although the number of these papers is limited, but surely destined to grow with time. The Quantra^®^ analyzer by Hemosonics is an example of one of these new VHAs. The Quantra^®^ VHA is based on the sonic estimation of elasticity via resonance (SEER), or sonorheometry. This is a technology that measures clot time and clot stiffness by using ultrasound to monitor changes in the viscoelastic properties of a whole blood sample during coagulation. Shear modulus is measured by ultrasound without the use of moving mechanical parts or direct contact with the blood sample (Figure 3) [27,78].

The Quantra^®^ system is a cartridge-based, fully automated, four-channel device. Currently, the QPlus^®^ cartridge is cleared by the FDA for adult patients and it is indicated for use in the perioperative setting of cardiac and major orthopedic surgery. A second cartridge, QStat^®^, intended for use in trauma and liver transplant surgery, is currently only available outside the USA. An important distinction between classical VHA technologies and SEER sonorheometry is that SEER sonorheometry is based on direct measurements of the clot’s shear modulus in rational pascal units, an objective parameter that describes the elastic properties of a solid material. It enables Quantra^®^ to provide a true assessment of clot stiffness [79]. Unlike TEG^®^ 5000 and ROTEM^®^ delta tracings, the Quantra^®^ analyzer organizes its test results in three different views: trend data, clot stiffness curves, and dial results. It shows very good correlation with equivalent metrics from other VHA systems [68,70,80,81,82]. It has gained clearance both in Europe and in the US and it is cleared by the FDA for use in cardiac and major orthopedic surgeries. A few studies have reported the use of the Quantra^®^ in coronavirus disease 2019 (COVID-19) patients [80,82,83].

The cartridge-based methodology of the TEG^®^ 6s and Quantra^®^ use dry reagents and are fully automated, providing greater reproducibility and simplified quality assurance for these POC tests [71,74,84]. The definition and clinical significance of the Quantra^®^ parameters are detailed in Table 3.

#### 2.5.4. ClotPro^®^

The ClotPro^®^ is a viscoelastic cup and pin method like the original cup and pin technologies of the TEG^®^ 5000 and ROTEM^®^ delta analyzers. With ClotPro^®^, the pin is stationary and the cup spins [65]. ClotPro^®^ has simplified the multiple pipetting procedures involved with the first generation ROTEM^®^ and TEG^®^ with the use of a standard pipette that contains pre-dosed reagents that activate on contact with the blood sample. This sample is deposited into the cup prior to the automatic initiation of the test run. The basic parameters are similar to those of ROTEM^®^ delta with the addition of specialized testing and the exception that the parameters are listed as assays. The assays include specialized tests that are dependent on specific activators and inhibitors. These tests are: the EX-test, activated by recombinant TF, which assesses the extrinsic coagulation pathway and its interaction with blood platelets in citrated blood; the IN-test, which assesses the effect of heparin and is sensitive to FVIII; the HI-test, the IN-test with heparin inhibition; the RVV-test, which is highly sensitive to direct oral anticoagulants (DOAC) and detects Factor Xa (FXa) antagonists in citrated blood; the ECA-test, which is sensitive to and detects direct thrombin inhibitors (DTIs), such as dabigatran; the NA-test, which is a non-activated test; the FIB-test, which involves the examination of the fibrinogen level and fibrin polymerization in citrated blood, and where platelets are inhibited by dual inhibition with cytochalasin D and a synthetic GPIIb/IIIa antagonist); the AP-test, in which fibrinolysis is inhibited in-vitro using aprotinin, a potent direct antagonist of plasmin, the effector protease of fibrinolysis; and the TPA-test, in which the recombinant tissue plasminogen activator (r-tPA) triggers fibrinolysis, permitting the in vitro detection of antifibrinolytic agents in citrated blood. The definition and clinical significance of the ClotPro^®^ parameters are detailed in Table 3 [27].

### 2.6. Emerging Technologies

The emerging technologies of POC hemostatic assays involve the use of micro/nano electromechanical systems, fluorescent microscopy, photoacoustic detection, microfluidics [85], and electrochemical sensing [86]. Examples of these new technologies are laser speckle rheometry [87,88], mechanical resonant frequency [89], ultrasonic deformation, and parallel plate viscometry [90]. Parallel plate viscometry is a compact system which could be adapted for prehospital areas such as emergency medical services or potentially, in far forward combat areas for goal-directed POC-guided resuscitation [90]. These assays remain an area of vigorous research with emerging clinical applications which are beyond the scope of this commentary [27,91,92,93].

### 2.7. VHA Limitations

Because of an inherent lack of contact with collagen (representing endothelial disruption) and the lack of variability in shear stress to assess arterial vs. venous systems in the cup and pin technology, the fluid mechanics of VHAs do not replicate vascular hemodynamics. A solution to the biomechanical differences (which are not present in VHAs) is a method of rapid multichannel micro-fluidic detection which has been described to address the lack of venous and arterial sheer forces on the endothelial surface. This ex vivo testing reflects intravascular hemostasis more accurately than the VHAs [94,95].

Other limitations of TEG^®^ and ROTEM^®^ are their inability to detect (due to the overwhelming effect of thrombin) specific platelet receptor abnormalities without the use of specialized testing [96,97]. In addition, the detection of the hemostatic competence of patients treated with DOACs, DTIs, or warfarin also requires specialized reagents and testing. This is because the standard TEG^®^/ROTEM^®^ parameters demonstrate poor sensitivity within drug concentrations at therapeutic ranges, in addition to poor sensitivity for trough samples, and an overlap with controls. For warfarin, a lack of sensitivity in detecting anticoagulation and a low correlation with thrombin generation assays renders the TEG^®^/ROTEM^®^ unsuitable for detecting the therapeutic efficacy for heparinized patients [10,98,99,100].

VHAs are not sensitive to von Willebrand disease (vWD). The activation of the von Willebrand factor (vWF) requires high shear rate forces and collagen. Since VHA assesses blood flow under low shear rate, which represents venous flow, the detection of vWD is not possible using standard VHAs. The literature regarding the utilization of VHAs in the assessment of vWD has not demonstrated robust applicability. However, there have been research modifications to TEG^®^ and ROTEM^®^ that attempt to address this limitation. Specialized TEG^®^, called ristocetin-enhanced TEG with Platelet Mapping^®^ (TEG/PM^®^), has suggested that the TEG^®^ 5000, as well as ROTEM^®^ delta assays with ristocetin thromboelastometry (RicoTEM^®^), can be used to assess vWD [101,102,103,104,105,106,107,108].

TEG^®^ without platelet mapping, ROTEM^®^ without platelet assays, and Quantra^®^ are unable to measure the results of antiplatelet therapies (e.g., clopidogrel and aspirin) on the level of inhibition of specific platelet receptor activities [71].

VHAs are not sensitive to the effects of FXa inhibitors (apixaban, rivaroxaban, edoxaban) or DTIs. Modified TEG^®^ and ROTEM^®^ systems have been described that measure the effects of FXa inhibitors, as well as DTIs [17,50,100,109,110,111,112].

TEG^®^ 6s global and Platelet Mapping^®^ cartridges, as well as QPlus^®^ Quantra^®^ cartridges, do not display the lysis parameter. Intensive care patients manifest significant correlation of the MA and R, substantial correlation in α angle and K, but poor correlation in LY30, as measured by the TEG^®^ 6S and TEG^®^ 5000 analyzers. Apart from LY30, the TEG^®^ 6S and TEG^®^ 5000 platforms appear to be interchangeable. This has important implications for use in clinical practice and multi-site research programs [66].

### 2.8. Viscoelastic Hemostatic Assay Guidance of Blood Component Therapy and Pro-Hemostatic Agents

The use of VHAs to guide BCT, pro-hemostatic agents, and anticoagulants for those on extracorporeal membrane oxygenation (ECMO) represents a conceptual application of precision-based medicine (PBM) whereby each patient’s hemostatic phenotype is defined by the VHA [113,114]. Then, resuscitation or anticoagulation is guided by changes in the individual patient’s evolving phenotype indicated by the VHA parameters. These changes in hemostatic phenotype can be rapid in all areas of bleeding or clotting and are known as moments of “phenotype switching” [17,20,38,113,115]. Targeted, goal-directed administration of BCT, guided by VHA POC testing, allows for a more accurate and personalized method of resuscitation [10,11,113,116,117,118].

#### 2.8.1. General Principles of VHA-Guided BCT, Hemostatic Adjuncts, and Anticoagulation

Tailored resuscitation or anticoagulation of patients with acquired coagulopathies can be achieved with the administration of pro-hemostatic adjuncts, BCT, or anticoagulants in response to the parameters of the VHA. A long CT/R value in hypocoagulopathic patients can be corrected with fresh frozen plasma (FFP) and prothrombin complex concentrate (PCC). For those patients anticoagulated with UFH, a reduction in UFH and/or reversal with protamine sulfate can correct the long CT/R value. Note that there is no standardized TEG protocol for protamine reversal and that these protocols vary by institution [119]. A prolonged CFT/K or decreased α-angle requires fibrinogen concentrates or cryoprecipitate. A narrow MCF/MA may be corrected with fibrinogen concentrate, cryoprecipitate, and platelets. Epsilon aminocaproic acid or tranexamic acid is given for an elevated lysis demonstrated by elevations of parameters such as CLI30 and LY30 [11,120,121,122]. Hypercoagulability in the post-trauma resuscitation period is indicated by a wide MCF (ROTEM^®^), wide MA (TEG^®^), a low CLI30/ML (ROTEM^®^), and a low LY30 (TEG^®^) [123]. When specific activators or inhibitors are added to the cup to identify specific disruptions in coagulation, such as platelet dysfunction or fibrinogen, tailored therapies may be given [11,31,36]. Sample VHA guidelines are reproduced in Table 4.

#### 2.8.2. Specialized Testing: TEG Platelet Mapping^®^ (TEG/PM^®^), ROTEM Platelet Analysis^®^, Isolated Platelet Dysfunction, Multiple Electrode Aggregometry (Multiplate^®^)

Platelet dysfunction as a manifestation of the early coagulopathy in cases of trauma is a recently described phenomenon. The methodology for determining platelet activation in circulating blood is an area of continued interest since the measurement of platelet activation at the luminal site of the clot does not reflect the platelet activation at the core of the clot at the site of the endothelial injury [125,126,127,128].

An improved survival rate has been demonstrated by epidemiological studies on those combat and civilian patients who received higher ratios of platelets than FFP and red cells [129]. As a result, in massive transfusion protocols, platelets are now given in the earlier rounds of transfusion. However, because ROTEM Platelet Analysis^®^, TEG/PM^®^, and Multiplate^®^ rely on completely different technologies, their place in guiding platelet administration in the cases of hemorrhagic shock and massive transfusion remains an area of active research (Table 5) [36,125,127,130]. Therefore, the clinical indications to administer platelets based on these assays remains in the early stages of development [126,131,132].

#### 2.8.3. VHAs Reflecting Anticoagulation

Unfractionated heparin activity can be effectively evaluated by VHAs. However, direct and indirect thrombin inhibitors, as well as low molecular weight heparin, cannot be assayed well with unspecialized VHAs. The addition of heparinase to one of the cups allows the VHA assays to quantify the heparin affect, and these assays are therefore used frequently in liver transplantation and cardiac surgery. Most recently, they have been used to monitor ECMO patients, as well as heparinized COVID-19 patients [36,114,133,134,135,136]. The assessment of non-thrombin-based platelet activation/inhibition can be assayed using TEG/PM^®^ and Multiplate^®^, which is often used in concert with ROTEM^®^. TEG/PM^®^ parameters of AA and ADP platelet receptor activity and Multiplate^®^ parameters have demonstrated a correlation between the percent of inhibition and the severity of the hemorrhage in bleeding trauma patients [22,36,41,125,127,137,138,139,140,141,142,143,144,145,146]. Moreover, ROTEM^®^ has demonstrated its use for the personalized titration of anticoagulation for patients with difficult coagulopathies. For example, for perioperative patients who require anticoagulation because of the risk of thrombosis, but who also have hypofibrinogenemia, ROTEM^®^ assists in the mitigation of both the bleeding and thrombosis [147].

## 3. Discussion and Conclusions

Compared to the historically delayed uptake in the use of VHAs, the recent National Institutes of Health and FDA statements, the major international society guidelines from hematology and critical care societies, and the meteoric rise in the number of related papers in the most recent literature since the advent the COVID-19 pandemic have revealed a changing landscape regarding the acceptance of VHAs in guiding the assessment of hemostatic competence in severely bleeding and septic patients [71,134,149,150,151,152,153,154].

Since early 2020, many papers from case reports to narrative and systematic reviews have been published regarding the use of VHAs in the treatment of COVID-19-associated coagulopathy (CAC). Initially, single case reports were often submitted as letters to the editor in order to accelerate the speed of publication. These case reports demonstrated the utility of VHAs to diagnose and treat CAC. In early 2021, narrative and systematic reviews subsequently revealed a common thread describing the reproducible changes in parameters induced by early periods of the cytokine storm. As a result, many prospective, retrospective, and single-center observational studies have been published using ROTEM^®^, TEG^®^, and most recently, Quantra^®^ and ClotPro^®^ devices, to study critically ill COVID-19 patients. [134,150,151,155,156,157,158,159]. In a little over a year, the rapid adoption of VHAs to guide prophylactic and anticoagulant therapy in patients with CAC contrasts starkly with the more than the 73-year evolution of the use of these tests in other areas of medical research and practice. This unique period in history called for the adoption of a type of scientific methodology that met the demands of an urgent need to discover valid forms of therapy for these patients. The use of VHAs to define a patient’s hemostatic competence along the pathophysiologic spectrum of coagulopathies that characterize CAC has allowed for the rapid deployment and application of precision-based medicine that defines each patient’s hemostatic phenotype [38,113,134].

During this period in the history of the rapidly evolving COVID-19 pandemic, large randomized controlled trials and Cochrane analyses are not sufficient to determine the utility of the adoption of new technologies that provide individualized therapies to patients to address their evolving hemostatic phenotypes [151,160,161]. Smaller clinical observations, based on a mechanistic rationale, proved useful during the early period of the pandemic for those patients who did not respond to the standard venous thromboembolism (VTE) prophylaxis recommended by many institutions. As a result, after a period of support for the full anticoagulation of severe patients, a high incidence of bleeding was noted, confirming smaller studies involving fully anticoagulated COVID-19 patients [136,162].

In an attempt to accelerate the pace and accuracy of scientific discovery, the rapid deployment of multiplatform RCT (mpRCT)-type studies have been undertaken regarding therapeutic anticoagulation in these patients [163]. These studies have shown that early anticoagulation, with intermediate or full anticoagulation prior to arrival in the intensive care unit, may prevent endotracheal intubation and ECMO, and may reduce mortality [163]. Additionally, one study has shown that VHAs demonstrated platelet-driven hypercoagulability and that some patients may benefit from both anticoagulation and antiplatelet therapy [119]. In this area, it has been suggested that the utilization of VHAs may increase the sensitivity of the monitoring of these patients before and during ICU admission. In none of these mpRCTs have VHAs been uniformly studied and analyzed [67,163,164].

The COVID-19 pandemic has elevated the need for rapid POC testing to gauge the COVID-19 patient’s hemostatic competence, thus ushering in accelerated utilization of the VHAs to diagnose and treat patients with CAC [23,134,135,150,151,153,165,166]. The hypercoagulopathy of CAC inversely mirrors the hypocoagulopathy of many other areas of clinical medicine where hypocoagulopathies can quickly evolve in the medical and surgical intensive care units, obstetrical units, and operating theaters. Relying on CCTs has proven difficult in managing these acquired coagulopathies [7,36,42,126,167,168,169,170,171]. Unlike the hemorrhaging hypocoagulopathic patient, COVID-19 patients most often present with hypercoagulopathic findings and clinical thromboses in all vascular beds as a reflection of the intensity of the cytokine storm [172,173].

Immuno-thrombosis has been described as the dysregulation of the crosstalk between coagulations and immunity [152,173,174]. Since the pathophysiology of CAC involves an endotheliitis which affects all systems, the concept of immuno-thrombotic crosstalk also occurs with the cross-hybridization of the research and clinical experiences of the many specialties which care for patients with endothelial dysfunction, specifically, those specialties that have observed the condition called Shock-Induced Endotheliopathy (SHINE) [175], which often presents with coagulopathies that are a function of immune-thrombotic dysregulation in trauma and sepsis [17,172,175,176].

CAC may be characterized as “upside-down trauma,” whereby COVID-19 patients present in a hypercoagulopathic state but may quickly evolve into a hypocoagulopathic state, opposed to progression of the typical trauma patient [151,173]. The index VHAs of severely bleeding trauma patients begin with hypocoagulopathy, manifested with long R/CT, flat alpha angle, thin MA/MCF, and increased LY30% and ML [10,20]. On the other hand, the index VHAs of hospitalized COVID-19 patients display a hypercoagulopathic presentation [134,150,177,178]. Hypercoagulability in CAC can quickly be modulated by either anticoagulant administration or by the innate changes caused by the vagaries of the cytokine storm. Eventually, these patients are rendered hypocoagulapathic either by anticoagulation or by the natural course of disease. As the cytokine storm recedes, these patients often develop an increased sensitivity to anticoagulation, causing them to bleed at varying times after the institution of anticoagulation. Hence, these patients are said to often “clot and bleed at the same time” [151,162,164,172,179,180,181,182].

Therefore, the monitoring of CAC patients can be quite difficult when the task has fallen to CCTs. As in trauma, adjunctive VHAs have also been used to guide therapies for CAC patients. This is due to the fact that CCTs provide inadequate data in severely hemorrhaging patients. CAC strikes the endothelium, influencing every aspect of anticoagulation from initiation to termination, so more information than what is provided by CCTs is needed [133,160,172]. Increased D-dimer levels have often been described in COVID-19 patients, but the half-life of D-dimer is approximately eight hours or longer in cases of compromised renal function and in elderly patients; this indicates that highly increased D-dimer depicts fibrinolysis that occurred at a point earlier in time rather than fibrinolysis that is currently occurring [178]. COVID-19 patients have also demonstrated a degree of fibrinolytic shutdown as manifested by the increasing thickness of VHA parameters, such as increased MA in TEG^®^ and MCF^®^ in ROTEM, whereby the SARS-CoV-2-mediated elevation of endogenous fibrinolytic inhibitors (e.g., plasminogen activator inhibitor-1 [PAI-1], thrombin activatable fibrinolysis inhibitor [TAFI], and alpha2-antiplasmin) leads to the increased resistance to clot lysis in COVID-19 patients [178,183,184,185]. Moreover, the capacity for thrombin generation has been demonstrated to be normal despite anticoagulation with heparin [184].

Because the endothelium possesses a surface area up to 7000 square meters (the surface area of a soccer pitch), the myriad of hemostatic phenotype changes that can occur in septic and hemorrhagic patients requires POC bedside testing that allows the clinician to adapt to the rapid changes in the hemostatic profiles and phenotypes of these patients [176]. The many interrelated mechanisms of immunologic and thrombotic dysregulation can now be monitored by many specialties at the patient’s bedside. The need to assay every aspect of the life span of a clot has resulted in a recent joining of forces among medical intensivists, anesthesiologists, trauma critical care physicians, transplant surgeons, obstetricians, emergency physicians, pathologists, blood banking specialists, and hematologists. They have gathered to share their experiences with not just the traditional post-operative and intraoperative associated coagulopathies of liver, heart, and trauma-induced coagulopathies, but also in areas where VHAs were less commonly used, such as sepsis-induced coagulopathy, obstetric hemorrhage, major medical hemorrhages, and now CAC [16,17,151]. Hence, every specialty has been confronted with the coagulopathies of COVID-19, and these specialties have reported their diverse respective experiences during the pandemic in the current literature. This meteoric expansion of the literature regarding VHAs and CAC has led to an increased adoption of POC VHAs, assisting clinicians at the bedside in the care of these patients with a significant heterogeneity of results. This heterogeneity is not merely a reflection on the multiple specialties who have been called upon to care for these patients, but also a reflection of the immune-thrombotic crosstalk at the level of the endothelium. This has resulted in a similar crosstalk among the many specialties who care for these patients [71,134,149,150,151,152].

The relatively rapid deployment of VHAs by specialists throughout the world from all areas of medicine has created a formidable foundation of research that allows for such an expansion of this POC bedside technique to monitor hemostatic competence [134,168]. As much as there is a spectrum of coagulopathy, a similar spectrum of inquiry is required that adopts both hypotheses-generating smaller studies and larger randomized control trials with the mpRCT functioning in between [67,151,164,172,173]. Hence, the use of both forms of inquiry will allow multiple specialists treating the endothelial immune-thrombotic derangement of many organs to approach the array of phenotypes of coagulation disorders in the endothelial beds. Thus, hematologists, medical intensivists, anesthesiologists, trauma critical-care surgeons, obstetricians, pathologists, blood banking specialists, as well as emergency physicians, can all contribute to broadening and filling the knowledge gaps within the spectrum of coagulopathies in the many medical specialties by using both CCTs and VHAs to monitor hemostasis across heterogeneous groups of patients. 

## Figures and Tables

**Table 1:** TEG^®^ 5000 and ROTEM^®^ delta “legacy device” parameters and reference ranges [7,27,35,37,38,42,43,44,45,46,47,48,49,50,51,52].

Parameter	TEG^®^ 5000 Analyzer ^1^	ROTEM^®^ Delta Analyzer
Clot initiation: the time from the test start to amplitude = 2 mm	R (Kaolin)/ACT (rTEG^®^) Kaolin: 4.6–9.1 min rTEG^®^: 86–118 s	CT INTEM: 137–246 s EXTEM: 42–74 s
Clot formation and clot kinetics: the time taken to achieve a level of clot strength, amplitude = 20 mm	K Kaolin: 0.8–2.1 min rTEG^®^: 1–2 min	CFT INTEM: 40–100 s EXTEM: 46–148 s
Angle of clot formation	α-angle Kaolin: 53–72° rTEG^®^: 64–80°	α-angle INTEM: 71–82° EXTEM: 63–81°
Maximum clot strength	MA Kaolin: 50–70 mm rTEG^®^: 52–71 mm MAff/CFF: 15–32 mm	MCF INTEM: 52–72 mm EXTEM: 49–71 mm FIBTEM: 9–25 mm
Lysis ^2^ TEG^®^: rate of clot breakdown 30 min after MA is reached. ROTEM^®^: the residual % of MCF amplitude when measured 30 min after CT (CLI30); the % reduction MCF amplitude at a given timepoint (ML)	LY30 Kaolin: 0–7.5% rTEG^®^: 0–7.5%	CLI30: 94–100% ML: <15%

Abbreviations: activated clotting time (ACT); clot formation time (CFT); clot lysis index at 30 min (CLI30); clotting time (CT); extrinsic thromboelastometry (EXTEM); fibrin-based thromboelastometry (FIBTEM); intrinsic thromboelastometry (INTEM); kinetics (K); lysis at 30 min (LY30); maximum amplitude (MA); maximum clot firmness (MCF); maximum lysis (ML); RapidTEG (rTEG^®^); reaction time (R); rotational thromboelastometry (ROTEM^®^); thromboelastography (TEG^®^); TEG functional fibrinogen (MAff/CFF). ^1^ For the TEG^®^ 6s and the TEG^®^ 5000, the manufacturer (Haemonetics, Braintree, Massachusetts) makes clear that the ranges are interchangeable, but that any difference in the values is a consequence of the Clinical and Laboratory Standards Institute (CLSI) methodology used for the TEG^®^ 6s values. Additionally, the manufacturer’s ranges are not globally prescriptive, as every laboratory must establish their own reference intervals [48]. ^2^ The values for clinically significant fibrinolysis vary greatly in the literature.

**Table 2:** Detailed descriptions of specialized TEG^®^ and ROTEM^®^ assays [32,36,37,53,58,59,60].

VHA	Test	Activator/Inhibitor	Significance
TEG	Kaolin	Kaolin, CaCl_2_	Contact activation.; similar information as aPTT; isolates the intrinsic pathway.
	rTEG^®^	Kaolin, TF, CaCl_2_	Clotting is accelerated by activation of extrinsic TF and intrinsic kaolin pathways; contact activation; roughly analogous to an ACT; information about coagulation kinetics initiated via contact activation alone is lost.
	HTEG^®^	Kaolin, lyophilized heparinase, CaCl_2_	Lyophilized heparinase neutralizes UFH; compared to kaolin TEG^®^ to assess the heparin effect.
	MAff/CFF	Kaolin, TF, Abciximab, CaCl_2_	Abciximab is a GPIIb/IIIa platelet receptor inhibitor that blocks the platelet contribution to clot formation; compared to kaolin TEG^®^ to assess the fibrinogen contribution to clot strength independent of platelets.
	Native TEG^®^	Calcium	Native whole blood sample analyzed following recalcification only; impractical for clinical use given long R.
ROTEM	INTEM	Ellagic acid, CaCl_2_	Tests clotting activation through the intrinsic coagulation pathway, FXII, FXI, FIX, FVIII, FX, FV, FII, and fibrinogen; sensitive to the heparin effect; similar information as aPTT.
	EXTEM	Recombinant TF, CaCl_2_, polybrene	Polybrene neutralizes UFH; tests clotting activation through the extrinsic coagulation pathway, FVII, FX, FV, FII, and fibrinogen.
	HEPTEM	Ellagic acid, CaCl_2_, lyophilized heparinase	Tests heparin and protamine sulfate effects in patients with high heparin concentration when compared with INTEM.
	FIBTEM	Recombinant TF, CaCl_2_, polybrene, cytochalasin D	Cytochalasin D blocks platelet activation; tests fibrinogen component contribution to clot stability; more sensitive to lysis.
	APTEM	Recombinant TF, CaCl_2_, polybrene, aprotinin/TXA	Tests fibrinolysis when performed together with the EXTEM.
	NATEM	CaCl_2_	Native whole blood sample analyzed following recalcification only; impractical for clinical use given long CT.

Abbreviations: activated clotting time (ACT); aprotinin thromboelastometry (APTEM); activated partial thromboplastin time (aPTT); clot formation time (CFT); clotting time (CT); extrinsic thromboelastometry (EXTEM); fibrin-based thromboelastometry (FIBTEM); heparin thromboelastometry (HEPTEM); intrinsic thromboelastometry (INTEM); TEG functional fibrinogen (MAff/CFF); non-activated thromboelastometry (NATEM); reaction time (R); rapid TEG^®^ (rTEG^®^); rotational thromboelastometry (ROTEM^®^); thromboelastography (TEG^®^); tissue factor (TF); tranexamic acid (TXA); unfractionated heparin (UFH).

**Table 3:** A description of the four standard VHA assay parameters and their clinical significance with the addition of the most recent tools for measuring POC clot dynamics with the TEG^®^ 5000, TEG^®^ 6s, ROTEM^®^ delta/sigma, Quantra^®^ QPlus^®^, and the ClotPro^®^ systems [18,27,53,59,65,68,69,70,71,72,73,74].

Coagulation Event	Main Contributor	TEG^®^ 5000	TEG^®^ 6s	ROTEM^®^ Delta/Sigma	Quantra^®^ QPlus^®^ QStat^®^	ClotPro^®^	Clinical Significance
Clot initiation	Coagulation factors	Reaction time (R), minutes	R	Clotting time (CT), seconds	CT	CT	A short R/CT/CTH time indicates a hypercoagulable state. A prolonged R/CT time indicates either hypocoagulability or the presence of an anticoagulant. A short CTH/CKH in the presence of a long CT /CK-R or CTR > 1.1.indicates the presence of heparin anticoagulation. Specifically, heparin and DOAC tests are available with TEG^®^ 6s, ROTEM^®^ sigma, and ClotPro^®^. ^6^
		Citrated Kaolin (CK) R-time, minutes	CK-R-time, minutes	INTEM ^1^ CT	CT	IN-test CT	
		rTEG^®^ Activated clotting time (ACT), seconds ^1^	Citrated Rapid TEG (CRT) ACT, seconds	EXTEM ^1^ CT	n/a	EX-test CT	
		Citrated kaolin-heparinase (CKH) ^1^	CKH ^1^	HEPTEM ^1^ CT	Heparinase Clot Time (CTH), seconds	HI-test RVV-test ECA-test NA-test	
		n/a	n/a	n/a	Clot time ratio (CTR)	n/a	
Clot kinetics: amplification	Fibrinogen	Kinetic time (K), minutes	K	Clot formation time (CFT), seconds	Fibrinogen contribution to stiffness (FCS), hPA ^2^	CFT	Angle reflects fibrin kinetics, including fibrin formation and cross-linking. FCS measures the direct contribution of stiffness generated by fibrinogen.
		ɑ angle	ɑ angle	ɑ angle	n/a	ɑ angle	
		Citrated functional fibrinogen (MA_ff_/CFF)	MAff/CFF ^1^	FIBTEM ^1^	n/a	FIB-test ^1^	
Clot stiffness: propagation	Fibrinogen, Platelets	CK Maximum amplitude (MA), mm	CK MA, mm	Maximum clot firmness (MCF), mm	Clot stiffness (CS), hPA ^2^	MCF	MA, MCF, and CS reflect platelet and fibrinogen contributions to the clot stiffness and full platelet potential under maximal stimulation by thrombin. PCS isolates the platelet contribution to clot stiffness.
		rTEG^®^ MA, mm	CRT MA, mm	EXTEM ^3^ MCF	Platelet contribution to clot stiffness (PCS), hPA^2^	EX-test MCF ^3^	
Clot stability: termination	Fibrinolytic enzymes and inhibitors, Factor XIII	Lysis at 30 min after MA, (LY30), %	LY30	Clot Lysis Index at 30/60 min after CT (CLI30/CLI60), residual % of MCF ^4^ Maximum Lysis (ML), % of MCF lysed during run	Clot Stability to Lysis (CSL), % ^5^	Clot Lysis Index at 30/60 min (CLI30/CLI60), % of MCF ^4^	Hyperfibrinolysis is suggested by increased clot lysis that starts within 30 min of clot formation.
		n/a	n/a	APTEM ^1^	n/a	AP-test ^3^	
		n/a	n/a	n/a	n/a	TPA ^3^	

^1^ See Figure 1 and Figure 3 and Table 1 and Table 2 for the definition of the parameters. ^2^ hPa, hectopascals are units of pressure used to assay clot stiffness in Quantra^®^. ^3^ See Section 2.5.3 and Section 2.5.4 in the text and Figure 3 for definitions of the Quantra^®^ and ClotPro^®^ assays and parameters. ^4^ For the purposes of this review, in ROTEM^®^ sigma/delta and ClotPro^®^, fibrinolysis is defined uniformly as the clot lysis index (CLI) ^5^ Measurement of Clot Stability to Lysis (CSL) is only available with the QStat^®^ cartridge. ^6^ Assays for DOACs (Factor Xa inhibitors and direct thrombin inhibitors) are in various stages of approval for commercial use.

**Table 4:** Proposed trigger values for the rTEG^®^ 5000 and ROTEM^®^ delta [32,38,39,50,116,123,124].

rTEG^®^ Trigger Value	ROTEM^®^ Trigger Value	Intervention
ACT > 128 s	EXTEM CT > 80 s	PCC/FFP
α-angle < 65° MAff/CFF < 11 mm	EXTEM α-angle < 63° FIBTEM CA10 < 7 mm	fibrinogen/cryoprecipitate
MA < 55 mm	MCF < 45 mm	fibrinogen/cryoprecipitate/platelets
LY30/60 > 7.5% ^1^ citrated TEG/r-TEG	EXTEM CLI30/60 < 82% ML > 15% ^1^	TXA/aminocaproic acid

Abbreviations: Activated clotting time (ACT); clot amplitude at 10 min (CA10); clot lysis index 60 min after CT (CLI60); clotting time (CT); extrinsic activator thromboelastometry (EXTEM); fresh frozen plasma (FFP); fibrin-based thromboelastometry (FIBTEM); lysis at 30 min (LY30); maximum amplitude (MA); TEG functional fibrinogen (MAff/CFF); maximum clot firmness (MCF); prothrombin complex concentrate (PCC); Maximum lysis after 30/60 min (ML30/60); rotational thromboelastometry (ROTEM^®^); RapidTEG^®^ (rTEG^®^); thromboelastography (TEG^®^); tranexamic acid (TXA). ^1^ The values for LY30/60, CLI30/60, and ML as markers for clinically significant fibrinolysis vary significantly in the literature.

**Table 5:** TEG/PM^®^ and ROTEM Platelet Analysis^®^: specialized assays and their clinical significance [58,148].

VHA	Assay	Reagents	Clinical Significance
TEG/PM^®^	Kaolin TEG MA	Kaolin	MA_CK_ parameter is a proxy for the maximum potential function of the platelets. Thrombin overrides inhibition via anti-platelet agents, thus kaolin-activated TEG^®^ samples will not exhibit their effects.
	Activator TEG MA	Heparin, reptilase, FXIIIa	MA_Activator_ represents the isolated fibrin contribution to clot strength. Performed alongside K-TEG^®.^
	TEG MA_AA_	Heparin, reptilase, FXIIIa, AA	Measures AA contribution of platelet activity to clot strength. Performed alongside K-TEG^®.^
	TEG MA_ADP_	Heparin, reptilase, FXIIIa, ADP	Measures the ADP/GPIIb/IIIa pathways’ contribution of platelet activity to clot strength. Performed alongside K-TEG^®.^
ROTEM Platelet Analysis^®^	ARATEM	AA	Determines GPIIb/IIIa and COX-1 receptor inhibition.
	ADPTEM	ADP	Determines GPIIb/IIIa and ADP (P2Y12) receptor inhibition.
	TRAPTEM	TRAP-6	Determines GPIIb/IIIa and thrombin (PAR-1) receptor inhibition.

Abbreviations: Adenosine di-phosphate (ADP); Adenosine di-phosphate thromboelastometry (ADPTEM); Arachidonic acid (AA); Arachidonic acid thromboelastometry (ARATEM); Citrated kaolin (CK); Kaolin-TEG (K-TEG^®^); Maximum amplitude (MA); Rotational thromboelastometry (ROTEM); Thrombin receptor-activating peptide-6 (TRAP-6); thrombin receptor-activating peptide thromboelastometry (TRAPTEM); thromboelastography (TEG); TEG^®^ Platelet Mapping (TEG^®^ P/M).

## Data Availability

Not applicable.
